# Perceptions of Therapeutic Intervention in Suicide Crisis Counseling in Experienced Korean Counselors: A Concept Mapping Study

**DOI:** 10.3389/fpsyt.2021.784710

**Published:** 2022-01-12

**Authors:** AeShil Park, Dongil Kim, HyeYun Gladys Shin

**Affiliations:** ^1^College of Medicine, Ulsan of University, Seoul, South Korea; ^2^Department of Education, Seoul National University, Seoul, South Korea

**Keywords:** suicide crisis counseling, experienced Korean counselors, therapeutic intervention, concept mapping, counselor perception

## Abstract

Within Organization for Economic Cooperation and Development (OECD) nations, South Korea has the highest suicide rate for which immediate prevention measures are sought including effective therapeutic counseling interventions. As such, the present study explored and examined experienced South Korean counselors' perception of therapeutic interventions for the prevention or delaying of completed suicide, using concept mapping methodology. The semi-structured interviews were provided to 15 study participants of experienced counselors having a minimum of 5 years of professional counseling career and at least 10 suicide crisis counseling sessions. A total of 77 statements were extracted with 8 major clusters: “Securing Safety,” “Active Advocacy for Client,” “Coping Skills Training,” “Conceptualization of Suicide Crisis,” “Emotional Identification and Validation,” “Empowerment,” “Counselor Self-Disclosure,” “Counselor Self-Awareness and Regulation.” From the results, the present study described unique findings in Korean counselors' perceptions of suicide crisis therapeutic intervention. Study limitations and future implications are further discussed.

## Introduction

From 2003 to 2019, South Korea continuously had the highest suicide rate among Organization for Economic Cooperation and Development (OECD) nations except for 2017 (1). That is, the average suicide rate in Korea indicates 24.6 persons in every 100,000 citizens, exceeding the global average of 11.5 persons for the same population (2). Investigating the nation's sociocultural background and suicide risk factors that are contributing to such a high rate is necessary, and research on distinguishing effective therapeutic counseling interventions for suicide prevention is essential.

Suicide is an unfortunate event varying by individuals, and it is worth speculating on sociocultural background and possibly related risk factors that may influence the exceptional suicide phenomenon in Korea. Considering the sociocultural background, Korea has fast advanced in individualism and capitalism together; however, rooted in Confucianism, interpersonal relationship-based communitarianism in its society is quite influential and important in its culture. In cultures that value individualism, individuals' wants and needs, rights, and diverse points of view are mutually respected, whereas communitarianism-based cultures accentuate common profits and interpersonal harmony (3). Especially according to the values of Confucianism affecting the Korean culture, conducting own duties in the family (4) and being a contributing citizen in the society are highly emphasized and suppressed (5). A communitarianism-based society can offer physical and psychological benefits to at-risk citizens when regulation and integration function well within the society; however, it can also negatively affect them when regulation and integration do not coherently function while undermining each citizen's individual values and overstressing the community benefits (6). Unfortunately, South Korea still experiences a certain level of instability and confusion between cultural communitarianism, Confucianism thoughts, and competitive individual values of western cultures due to its abrupt socioeconomic changes in the past decades (5), portraying an “anomic” state as decribed by Durkheim in 1987 (7). An anomic state is a pathological state with a weakened relationship between an individual and society. Between 1997 and 1998, the suicide rate in Korea suddenly increased due to the economic crisis (8, 9), and the rate has been continuously on the rise; the interpretation was that those individuals chose to commit suicide for feeling isolated and as a “burden” to others in weakened social integration (10).

In partial explanation for choosing suicide for the feelings of isolation and “burden” to others, Interpersonal Theory of Suicide (IPTS) has gained attention (11), and empirical research has been introduced with Korean participants (12–16). According to IPTS, suicide occurs when one's interpersonal desires and the capability for suicide coexist. Interpersonal desires consist of two aspects of “thwarted belongingness” and “perceived burdensomeness.” Thwarted belongingness indicates a frustrated state of wanting to belong, loneliness and deficiency of reciprocal relationship; perceived burdensomeness indicates one's self-hatred with a sense of being a burden to others. IPTS underscores that thwarted belongingness with perceived burdensomeness along with having capability for suicide (e.g., high tolerance toward physical pain, fearlessness in death) together result in committing suicide. A survey study conducted on 402 college students (178 men and 224 women) indicated significant effects of self-worthlessness and a sense of burdensomeness to others on college students' suicide rates (12). Similar results were found in another study on 331 young adults of 19–35 years old (16).

When western (USA, Great Britain, Canada) and Asian (Hong Kong, Taiwan, China, Thailand) groups were compared, the common risk factors for both population groups were physical illness, mood disorder, substance abuse, and alcohol abuse (17–22). Studies on Korean clients having suicide attempts or cases of committed suicide are rare to find. The Korean suicide group of 122 and the comparison group of 40 cases were compared for suicide group characteristics (23). A psychological autopsy study with case-control design uses an odds ratio to examine the differences found in control and case groups, and its results indicated that the Korean suicide group had a significantly lower unemployment rate compared to the same population group in western studies. In contrast, disconnection/withdrawal from social activities and abrupt interpersonal atrophy were major indicators in this suicide group. In addition, self-injury and previous suicide attempts were reported as the most dangerous factors that are absent in the comparison group. Koreans were reported as being more influenced by their family and loved ones' suicide than those in North America and Europe (23). Experiencing a serious level of stress due to having problems with a domestic partner and failure in business also had an effect on suicide. A study on 537 patients with suicide attempts at the hospital emergency rooms revealed that the young women population had a significantly high rate for repeated attempts (24). Within the repeated suicide attempts group, the single-households (e.g., divorced, separated, and deceased partner) had a higher rate of suicide and a higher ratio of interpersonal problems (23). Those who have attempted suicide are found to have fewer problem-solving skills and alternative strategies compared to the comparison group (25).

Different cultures have different factors and approaches to effective counseling (26, 27). Some of the characteristics of Korean counselors were compared to those of the USA, Norway, and Germany in a study, and the results demonstrated that only 5% in the western countries practiced counseling without a particular theoretical framework while 34.5% of Korean counselors practiced counseling without a theoretical framework (28). The interpretation of this result includes that limited applicability of the western theoretical framework can be carried on to Korean clients for the cultural, societal differences, and the study advocated that culturally fit counseling therapies should continue to develop (28). According to such needs, effective counseling factors in Korea were previously researched and the factors were “considering the client as own family member,” “applying private/emotionally attached relationship,” and “high involvement and active responses” as being effective factors reported by the Korean counselors (26). Advice or instructional interventions were found with a larger effect than in the western countries, and the clients' expectations included instructional, authoritative, short-term, and problem-oriented approaches (26, 28, 29). Korean clients tended to recognize counselors as competent when the counselors helped them express their inner feelings, in contrast to the expectation for the encouragement of rational objectivity (30, 31). Effective counseling factors in Korean counselors further included being aware of, understanding inexpressiveness on inner problems, patiently waiting in the initial stage, and realization of contrasting manifestations of the client's inner and outer expressions (26, 32). Additionally, Korean clients tended to consider effective counselors as exemplary role models and nurturers who can hold and withstand their negative feelings (29, 32, 33).

Knowing the helpful interventions in a counseling process for at-risk clients is valuable for suicide prevention education. The counseling process refers to both behavioral and verbal interactions between the client and counselor and their internal experiences from such interactions (34, 35). In contrast to the empirical and expert-derived studies on suicide crisis counseling competency in the western countries (36–38) and the studies on the client's recognition of effective counseling intervention in suicide crisis counseling (39, 40), Korea has almost no previous research on counseling intervention and its contents. This reflects the country's relatively short history in counseling and the clients' tendency toward feeling reluctant and embarrassed about publicly talking about their counseling experience (41); this cultural tendency particularly makes suicide crisis counseling research especially difficult to conduct. In this respect, speculating viewpoints of the counselors with suicide crisis counseling experience can offer promising possibilities as a path to acquiring effective therapeutic interventions.

Especially, experienced counselors can provide meaningful insights through their developmental phases with a qualitatively structured complexity. Using a small number of concepts, they can build and coherently conceptualize cases (42), are able to quickly discern similar patterns from interrelations between complex concepts (43), and are able to effectively process inconsistent and contrasting information from their own procedural memory (44). As a result, they are capable of building effective strategies by transferring the client's unstructured problems into structured concepts for problem-solving. This is an essential clinical competency in suicide crisis counseling in which many ambiguous situations rise for sudden, firm actions and successful strategies as for the most appropriate solutions (45).

For such reasons, this study has two purposes. First, the study aimed to identify therapeutic interventions in suicide crisis counseling perceived by experienced Korean counselors. Emphasis on the consideration of the sociocultural context in counseling has been accentuated on many occasions by previous research (46, 47), and suicide-related risk factors, protective factors, and adequate therapeutic interventions vary by culture (48). Speculating experienced counselors coping strategies and counseling techniques with suicide crises in the Korean context is worthwhile for possible prevention in the future. Second, this study aimed to develop a cognitive map of the counselors' perceived interventions through visual representation. Understanding the representation of the clustered interventions derived from the concept mapping is expected to contribute to (1) educating similarly sorted interventions considered therapeutic and (2) prioritizing important interventions.

## Methods

**Figure 1:**
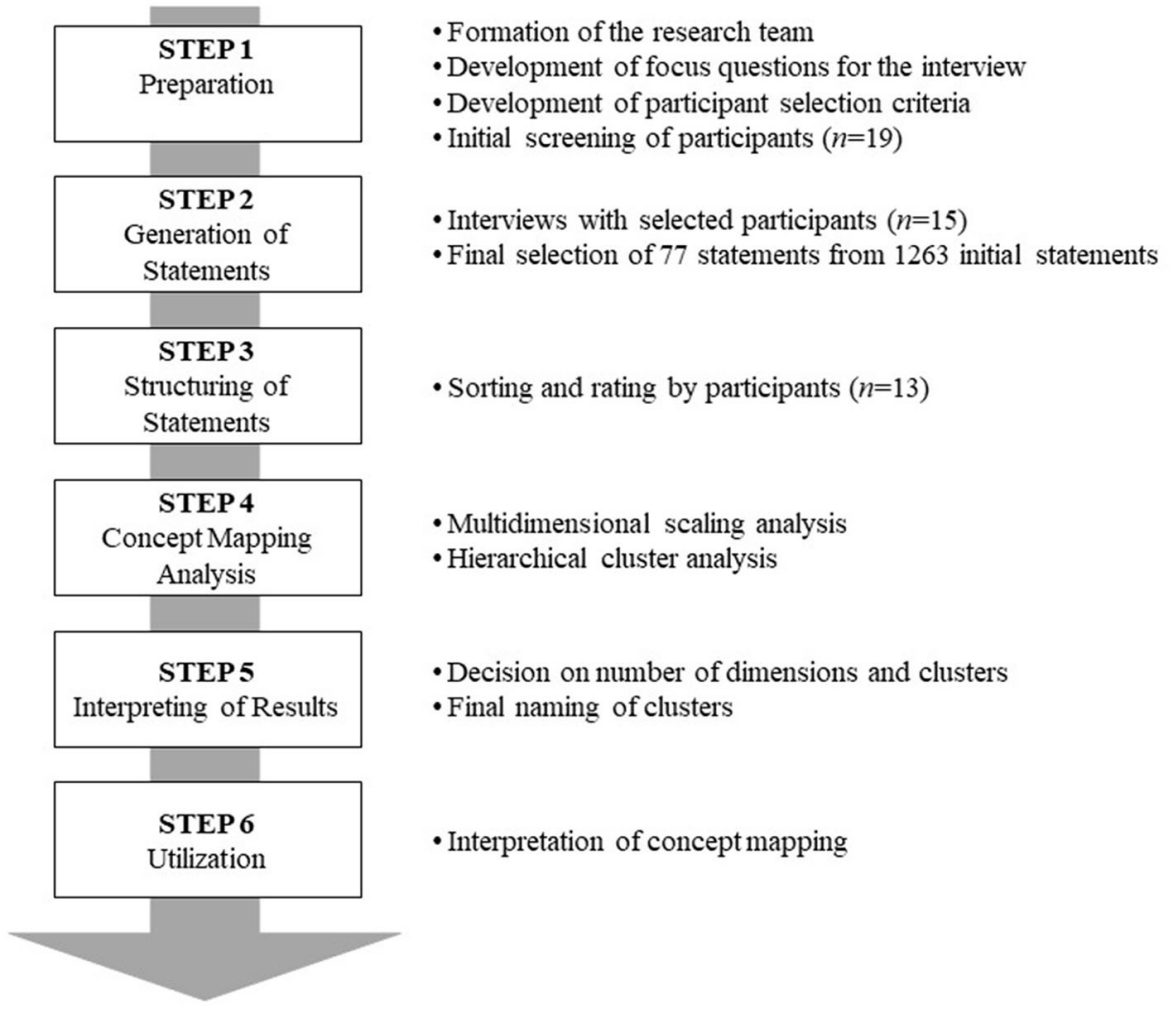
Concept mapping process-results.

The present study utilized the concept mapping methodology (49) to identify therapeutic interventions perceived by the counselors in suicide crisis counseling and to explore the structure of the interventions. Concept mapping consists of six procedural steps and is depicted in Figure 1.

### Participants

#### Selection Criteria for Cases of the Suicide Crisis

Wenzel, Brown, and Beck defined suicide crisis as “a discrete, intense episode of suicide ideation accompanied by suicidal desire, a suicide attempt, or other suicide-relevant behavior” (50). However, terms related to suicide are not too refined, resulting in the mixed usage of “self-injury without suicidal attempts,” “suicidal attempts with intention,” and “completed suicide” for the general term “suicide” even among many professionals (50). In Korea, mutually agreed measurement (i.e., clinical scale) in suicide crisis counseling settings is absent. Therefore, this study defined the term “suicidal crisis” as “clients with suicide attempts with suicide intention” as the client's intention has been inspected as the crucial variable in suicide attempts (51). Also, suicide attempts in one's history have been the most predictive variable for later completed suicide, and it has been a strong indicator of increasing the risk of actual suicide with more fatal methods at a later stage (23, 24, 52–55).

#### Selection Criteria for Experienced Counselors

The criteria for experienced counselors in suicide crisis counseling had to be established. Since South Korea has a relatively short counseling history, several psychiatric doctors started psychological therapy in 1957 upon returning from the Korean War (56), and its history has only 20 years of counseling profession by general counselors, clinical counselors, social workers, and medical doctors under the provisions of Mental Health Care Act in 2000. Because of dissimilarities along with some existing similarities, the western nations and Korea may not place the same criteria to be applied; the present study attempted to derive the definition of experienced counselors from research findings done by Korean literature in the suicide crisis counseling field. First, in order to meet the operational definition, the participant's whole counseling career had to be 10 semesters (5 years) or more as in the studies by Kim and Moon (57) and Son and Kim (58). Second, a basis for a suicide crisis counseling career had to be established for a certain duration of suicide crisis counseling to be an “experienced” counselor; however, no bases or criteria can be found according to previous research in Korea. Hence, as an alternative, this study focused on success factors found in phone-based suicide crisis counseling studies (59–61) and learned that continuing with suicide crisis counseling is critical because continuation itself signifies the prevention and delay of completed suicide. In a typical clinical setting, 10 sessions of counseling are a common unit of the session number, and this is also the minimum number of sessions for reliable efficacy in 50% of the clients (62). For this reason, this study defined “experienced counselor” with at least 10 completed sessions of suicide crisis counseling as the minimum requirement. In summary, the selection criteria for experienced Korean counselors of suicidal crisis counseling included counselors (1) with a minimum of 5 years of counseling career, (2) having the clients with “suicide attempts with a suicide intention,” and (3) having a minimum of 10 completed sessions.

#### Recruitment Process

Generating of statement process was done through both voluntary and referred participants. First, for the recruitment of voluntary participants, the study's purpose and information were posted on Korean Counseling Association (KCA) and Korean Counseling Psychological Association (KCPA). With approval by Institutional Review Board (IRB), the posting started on June 24, 2019, and recruitment ended on September 30, 2019. The purpose of the study, agreement for confidentiality, and possible risks that may arise during upcoming interviews were shared with the participants. The next step included contacting related personnel at counseling agencies, counseling-related university professors, phone calls with some previous interview participants, emails, and direct visits through which the clear purpose of this study was explained and any referrals for participants were requested. After receiving referrals, voluntary participation was guaranteed by first disseminating the recruitment letter and then receiving the participants' written agreement to participate. This purposive sampling process is useful when recruiting professionals on relevant topics or when finding participants is difficult (49).

**Figure 2:**
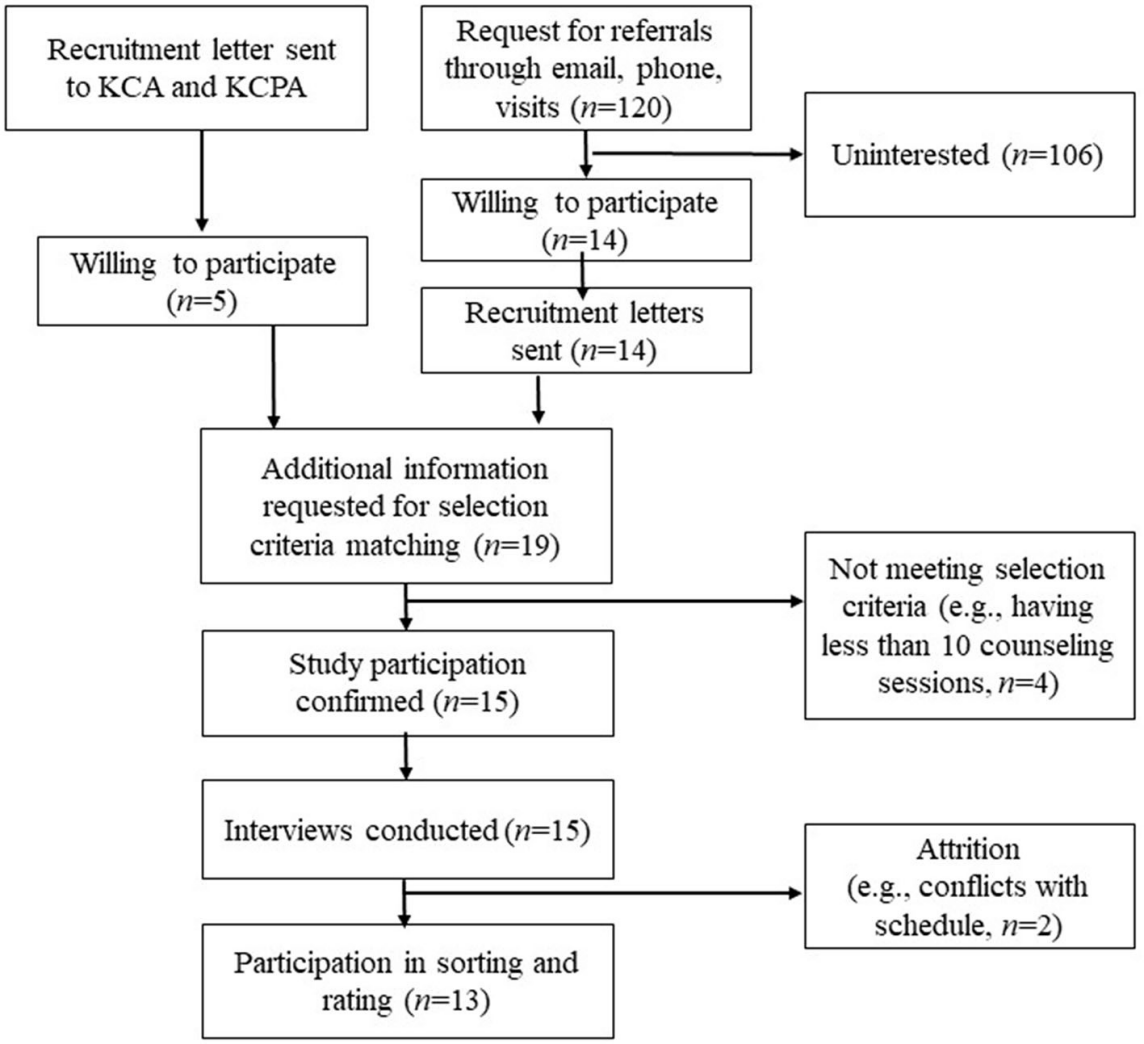
Participant process flow chart.

In the initial stage of recruitment, 19 counselors volunteered, of whom four were excluded because their number of counseling sessions did not meet the minimum requirement of 10 sessions (see Figure 2). Finally, 15 (three men and 12 women) counselors participated in this study, ranging from 34 to 50 years old (*M* = 40.13, *SD* = 5.80). Three of them had a master's degree, nine were currently in the Ph.D. program, and three of them already graduated from a doctoral program. One participant was employed at a corporation, four participants were practicing at clinics, five were working at the universities, one was working for the national public sector, and four were working at private counseling centers. Ten of the 15 participants owned a supervisory license and the remaining five participants owned a professional counselor license by the Korean Counseling Association (KCA) or Korean Counseling Psychology Association (KCPA), and their average counseling career was 9.8 years. Thirteen of them also participated in sorting of statements and importance rating (Step 3) in the later process of the study.

### Procedure

#### Step 1: Preparation

In step 1, the research team developed the focus questions for the interview. The focus questions were, “For the suicide crisis counseling clients, what do you believe are some therapeutic interventions?” After IRB approval was attained, participants were finally recruited. Kane and Trochim suggested a minimum of 10 study participants and emphasized statement generation should be sufficiently collected (49).

#### Step 2: Generation of Statements

The primary researcher conducted the interview with 15 participants. The interviews lasted between 50 and 90 min with an average time of 70 min. Interview materials were transcribed exactly following the participants' verbal expressions. In line with guidelines by Kane and Trochim (49), two doctoral students with extended suicide crisis counseling experience with an average of 11 years in career made a research team with the primary researcher and extracted therapeutic intervention lists. Any disagreements were resolved after a thorough discussion by comparing extracted intervention variable lists. A total of 1,263 statements were extracted from the interviews. The average number of statements was 84.2, reflecting 55–137 per participant. For the review of the adequacy of interview materials to true statements, transcriptions and statement items were sent to 15 participants by email or in person for verification and confirmation.

Kane and Trochim accentuate that for concept mapping to be successfully conducted, the number of statements and accuracy of the statements were the most important factors (49). Therefore, while maintaining brainstormed ideas, reducing the number of statements is necessary for idea synthesis processing. Although no concrete agreements were made on the most appropriate number of statements, Kane and Trochim recommend not to exceed 100 statements (49). In the first phase, the grouping was based on the conceptual area of intervention similarity. In the second phase, three research team members gathered to judge if the statements were just for therapeutic interventions, whether to keep the same grouping or to sort into a different grouping. With the guidelines by previous research (63), the synthesis process included only those statements found in common by at least two participants. In the third phase, only representative statements in sorted groups were generated. As a result, 85 representative statements were extracted. Then, for the evaluation of clarity and comprehensiveness, interview participants together discussed and exchanged the clarity and comprehension level of the statements. The research team edited once more after receiving feedback from those participants. A total of 77 statements were finally chosen.

#### Step 3: Structuring of Statements

On those 77 statements as therapeutic interventions, 10 x 3 cm cards were made and distributed to 13 participants for the grouping of homogeneous piles based on their conceptual similarities. In doing so, they were specifically advised not to make one card as a group or all of the cards together as a group and further advised to write an appropriate title for each pile of cards. After the sorting, the participants were asked to rate 1–7 on the scale of importance (1 = not at all important, 7 = extremely important) for each statement as the therapeutic intervention. Due to personal reasons, only 13 participants participated in the sorting and rating process.

#### Step 4: Concept Mapping Analysis

The following three steps of data analysis were implemented for concept mapping. In the first step, a similarity matrix was developed based on the results of 13 participants conducting the sorting. When the statements were grouped the same, the statement was coded as 0; when the statements were grouped differently, the statement was coded as 1. As per the number of participants in the study, 77 x 77 similarity matrix was formed with a combination of 13 piles to produce Group Similarity Matrix (GSM). In the second step, based on the group similarity matrix, the data was converted into a dissimilarity matrix using SPSS version 24, followed by multidimensional scaling analysis (ALSCAL). Using the results, the number of dimensions and meanings were analyzed for the therapeutic interventions in suicide crisis counseling. In the third step, the coordinate values derived from multidimensional scaling analysis were used for hierarchical cluster analysis (using Ward's method).

#### Step 5: Interpreting of Results

In step 5, the research team decided on the most meaningful number of clusters, and the participants' naming of the clusters was reflected and are explained more in detail later in the results section.

#### Step 6: Utilization

In step 6, the statements and clusters were presented in a two-dimensional graph, and the important topics of therapeutic interventions were deduced. More explanations are to follow later in the results section.

### Testimonial Validity

The concept mapping method has the strength of minimizing subjective viewpoints of the researchers (64) while reflecting participants' awareness of the occurrence through the research procedure (65, 66). Testimonial validity was designed to reflect participants' intentions during the process. First, the participants were contacted by email for reconfirmation of their responses on therapeutic interventions upon extraction. Second, a final list of 77 statements, along with the evaluation's collection of basic statements, was forwarded to the entire participants, and their feedback on the statements was collected. Lastly, during the process of naming the clusters from the sorting, the participants' word choices and suggestions on sorting criteria and topics were reflected.

## Results

### Multidimensional Scaling (Step 5)

In the multidimensional scaling analysis (ALSCAL), stress value is produced and used for the decision of dimension numbers. Kane and Trochim (49) state that “Stress measures the degree to which the distances on the map are discrepant from the values in the input similarity matrix” (p. 97). Kane and Trochim (49) assert 0.205–0.365 as the adequate range of the stress value. The sorting results were made into a similarity matrix and the multidimensional scaling analysis was conducted. From the results of the analysis, the associated stress value for the two dimensions was 0.31 (*R*^2^ = 0.49); the three dimensions was 0.21 (*R*^2^ = 0.64); the four dimensions was 0.15 (*R*^2^ = 0.75); the five dimension 0.13 (*R*^2^ = 0.79). Many researchers suggest that a two-dimensional model is ideal because a three-dimensional model can cause perceptual distortion although it can be visually presented (67). This study also chose a two-dimensional model as the most adequate model for the reason that a three-dimensional model was difficult to visually present after examining both two- and three-dimensional models within the valid stress value.

### Hierarchical Cluster Analysis (Step 5)

The hierarchical cluster analysis was conducted using two-dimensional model coordinate values from 77 statement dissimilarity matrix. The guidelines by Gol and Cook (68) were followed to determine the number of clusters. First, 13 participants sorted 4–18 groups for the 77 statements on the similarity rating (*M* = 12.0, *SD* = 4.38). Second, each cluster's conceptual clarity, similarity within a cluster, and inter-cluster distinctions were considered. Using Ward's method dendrogram, 2–8 clusters were compared for the final decision of eight clusters. Subsequently, after carefully considering the overall structure of distinctive features of different clusters and the final statement contents in all cluster areas, each cluster was named. The participants' feedback on the naming of the clusters was reflected in this step. Cluster 1 is named “Securing Safety,” Cluster 2 as “Active Advocacy for Client,” Cluster 3 as “Coping Skills Training,” Cluster 4 as “Conceptualization of Suicide Crisis,” Cluster 5 as “Emotional Identification and Validation,” Cluster 6 as “Empowerment,” Cluster 7 as “Counselor Self-Disclosure,” Cluster 8 as “Counselor Self-Awareness and Regulation.” The statements for each cluster are presented in Table 1.

**Table 1:** Statement by cluster on therapeutic interventions.

**Cluster/statement**	***M***	***SD***
**Cluster 1: Securing Safety**	5.59	0.70
01. Flexibly modify counseling frequency and time for crisis management.	4.62	1.33
03. When urgent, prioritize crisis management rather than rapport building.	5.85	1.21
11. When the client's functional level is low, focus on his/her safety and delaying the crisis.	5.62	1.26
19. Buy time by initially delaying suicide.	5.85	1.14
24. Work on improvement of problems with the client's present functional level so as to lower the crisis.	6.00	1.00
28. Provide emergency contact number in case of emergency.	6.46	0.78
29. Firmly state about counselor's stance opposing suicide.	4.46	1.51
31. Inform the client's (legal) guardians of the crisis and educate adequate coping skills for the crisis.	6.23	0.83
35. Inform what cannot be done during counseling, and discuss self-coping strategies.	5.15	1.57
36. If need be, recommend appropriate medication and steadily monitor the medication progress	5.77	0.83
37. Yield the client's promises on not committing suicide attempts.	5.15	1.41
44. If danger is detected, recommend hospitalization and support with the process.	5.69	1.32
51. Inform on suicide-related confidentiality and its limitations, and discuss necessary steps.	6.23	0.83
59. If urgent, check the client's safety first and help the client stabilize.	6.38	0.77
60. Consider potential influences to the client when hospitalization stops counseling sessions.	4.62	1.26
65. Build safety plans with the client, and, when necessary, counselor takes the lead in the process.	5.38	0.96
**Cluster 2**: **Active Advocacy for Client**	5.27	0.85
09. In order to unnecessary confusion, use the clear and easy-to-understand language.	4.77	1.59
12. Even after counseling sessions have been terminated, periodically check for safety.	4.23	1.59
17. When exploring suicide attempts/impulsive episodes, neutrally react upon facts rather than emotionally responding.	5.38	0.77
39. Find out social network support and help establish such support system.	5.38	1.04
47. Ask for help through counselor's affiliation (e.g., session extension, adjusting on sliding scale, substitute counselor when absent, etc.).	5.77	0.83
53. Be self-aware of counselor's intuition regarding the client's suicide risks.	4.92	1.38
55. Inform on resources that the client can use when in need of help.	5.38	1.26
63. Discuss about counseling termination timing and process for the client's various emotions related to reliance on the counselor.	5.69	0.95
77. Continuously monitor for suicide ideation and impulsivity.	5.92	0.86
**Cluster 3: Coping Skills Training**	5.27	0.91
05. Assist with acquiring effective coping skills for handling relationship conflicts affecting client's suicide crisis.	5.15	1.14
45. Inform alternative options to be replaced for suicide.	4.85	1.57
48. Help reduce self-destructive behaviors (e.g., alcohol abuse, dangerous sexual activities).	5.31	1.32
52. Help maintain simple ways of living (e.g., eating well, washing, sleeping, walking).	5.38	1.12
68. Help maintain regularity in life academically and/or in career.	5.38	1.50
72. Inform clear consequences of problem behaviors.	5.00	1.00
73. Explore self-injury process and discuss with the client about the ways of reducing it.	5.08	1.04
76. Inform specific behavioral steps for coping well.	6.00	0.82
**Cluster 4: Conceptualization of Suicide Crisis**	5.63	0.73
02. Evaluate protective factors of client's suicide risks.	5.77	1.24
07. Explore what suicide triggers mean to the client's life.	5.62	1.19
08. Check core beliefs of suicide attempts/impulsivity.	5.92	0.95
10. Understand client's weak functions and resourceful positive aspects.	5.08	1.44
15. Explore client's childhood background that may have effects in suicide ideation/impulsivity/ attempts.	5.08	1.19
32. Understand client's suicide crisis as the comprehensive manifestations of emotional, cognitive, physical and behavioral dimensions.	6.23	1.09
67. Explore carefully on the process from the triggers of suicide to attempts/impulsivity	5.54	1.05
69. Find out personal history, diagnosis and environmental factors that increase suicide risks.	5.46	1.13
75. Help client become aware of the reason and intention of suicide attempts.	6.00	1.00
**Cluster 5: Emotional Identification and Validation**	5.54	0.58
16. Name and identify the client's feelings behind suicide ideation/impulsivity/attempts and stabilize such feelings.	5.77	1.09
18. Observe the client's emotional state	5.38	0.96
26. Explore the client's feelings regarding suicide impulsivity/attempts.	5.69	1.38
33. Understand and sensitively respond to the prominent emotions (e.g., having a devastated feeling of left alone) that brought to the suicide crisis.	5.85	1.14
50. Listen attentively and stay on the contents and emotions throughout repeated suicide impulsivity and attempts.	5.62	0.96
25. Assist in expressing negative feelings concretely.	5.77	1.24
41. Lead the client not to avoid fearful feelings, but to have him/her be aware of, admit and stay with such feelings.	5.38	0.96
57. Use various methods (e.g., imagery, drawing) to help the client express his/her inner being.	4.54	0.78
66. Help and understand the client's ambivalent feelings toward death.	5.38	0.77
71. Assist the client to be aware of own desires or hopes related to own fearful emotions.	5.92	1.04
38. Be present with the client for his/her pain and dealing with his/her devastation.	6.31	0.63
42. Recognize the client's psychological and physical pains throughout suicide attempt.	5.08	1.19
58. Understand and empathize the fact that the client's desire to die does not go away easily.	5.23	1.01
61. Understand and support difficult feelings underlying the client's unwillingness to cooperate.	5.31	1.11
74. Empathize and validate the client's pains bad enough to want to commit suicide.	5.85	0.90
**Cluster 6: Empowerment**	5.15	0.92
13. Help the client constructively make his/her own meanings of life.	4.77	1.59
23. Try to find the client's wants and help become hopeful about them.	5.08	1.04
49. Recognize client's strengths and resilience for being alive at the moment.	5.69	1.11
56. Recognize and encourage client's small changes.	5.46	1.45
64. Reframe the existing problems from the positive perspective.	4.62	1.04
06. Obtain client's cooperation on intervention after establishing a reliable rapport.	5.15	1.28
14. Be a good object and reparent according to his/her developmental stage.	5.15	1.41
27. Help the client eventually face his/her own problems objectively without being controlled by the client and wait appropriately.	5.46	1.45
30. Treat the client with value and accept with unconditional positive regard	5.46	1.56
40. Sensitively seek intervention points with patience for good changes in the depressed and helpless client.	5.38	1.19
43. Support first even when the client crosses the boundaries with the counselor.	4.46	1.27
**Cluster 7: Counselor Self-disclosure**	4.91	1.08
20. Self-disclose counselor's humane feelings from listening to client's suicide impulsivity/attempts	4.77	1.09
21. Express that the client is meaning and important person to the counselor.	5.31	1.25
22. Tell the client that his/her death has an influence on the counselor.	4.38	1.45
34. Express “warm welcome” upon the client's return.	4.92	1.32
54. Deliver to the client the message the counselor wishes him/her to stay alive.	5.15	1.63
**Cluster 8: Counselor Self-awareness and Regulation**	5.77	0.86
04. Expand counselor's viewpoint on useful interventions through handling counselor's own difficulties through consultation/supervision/individual counseling	6.00	1.41
46. Endure overwhelming and negative feelings counselor experiences during suicide crisis counseling.	5.92	1.38
62. Recognize counselor's own negative feelings toward the client (e.g., anxiety, anger, frustration, helplessness, etc.)	5.77	0.83
70. Be careful not to intervene for the purpose of getting rid of counselor's own anxiety.	5.38	1.50

### Interpretation of Concept Mapping (Step 6)

**Figure 3:**
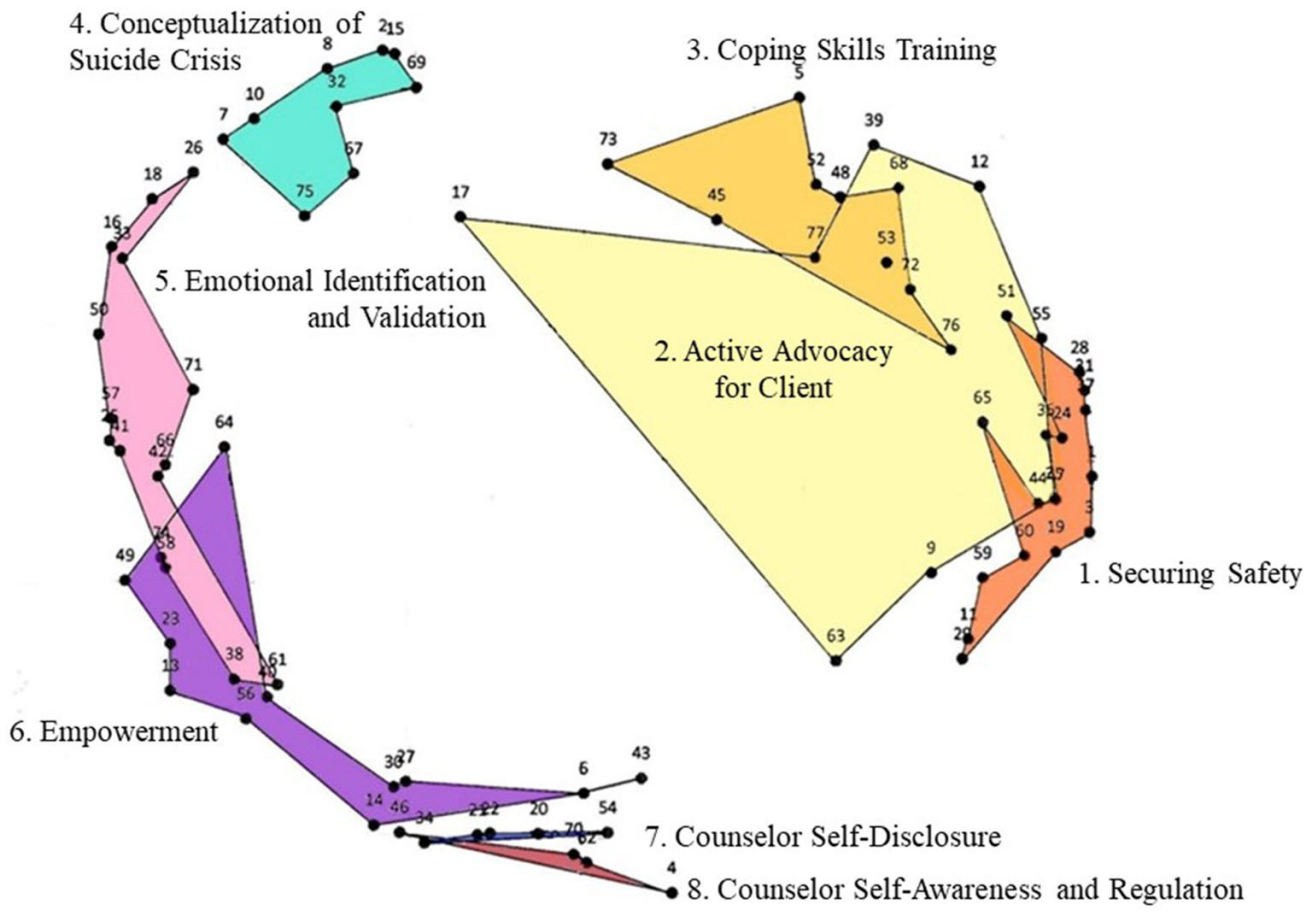
Concept map of therapeutic interventions of suicide crisis counseling.

Effective therapeutic interventions in suicide crisis counseling perceived by the participants are presented (see Figure 3). The distance between clusters indicates conceptual similarity (63). That is, the closer the distance, the more similarly do the participants recognize the clusters. For instance, Cluster 1 (Securing Safety), Cluster 2 (Active Advocacy for Client), and Cluster 3 (Coping Skills Training) are recognized with a high level of similarity by participants. Situated on the right, these three clusters are composed of contents of counselors' active and instructional interventions to the clients. In particular, counselors recommend hospitalization or medication for the client's safety and further collaborate with their legal guardians. The counselors additionally educate on alternative behaviors and techniques to reduce self-destructive behaviors along with actively seeking assistance from related institutions and connecting the clients to social resources available, advocating for the clients. Situated on the top, Cluster 4 (Conceptualization of Suicide Crisis) consists of identifying the risk and protective factors necessary for understanding the client's suicide crisis and helping the client realize self-intention for suicide attempts. Situated on the left, Cluster 5 (Emotional Identification and Validation) displays interventions of the counselor's endurance and willingness to patiently wait for the client's painful feelings to be expressed while empathizing with such feelings. Situated on the lower left, Cluster 6 (Empowerment), Cluster 7 (Counselor Self-Disclosure), and Cluster 8 (Counselor Self-Awareness and Regulation) consist of interventions deeply involved in the internal variables of the clients; the counselors related to the clients through truthful and therapeutic relationships to help them attain hope and more positive reconstruction in life. The counselors self-disclose on many aspects and express that the clients' existence is meaningful and important while realizing their own fear, suppression, overwhelmingness, and readjust accordingly to meet the client's therapeutic needs and provide adequate intervention.

As Table 1 illustrates, the average rating of importance for all 77 statements was 4.23, above the medium importance of four points. The statement indicating most importance for therapeutic intervention in suicide crisis counseling was “28: Provide emergency contact number in case of emergency” followed by “59: If urgent, check the client's safety first and help the client stabilize,” marking 6.46/7.00 and 6.38/7.00, respectively. Other items scoring higher than 6.0 of importance were Statements 28, 59, 38, 31, 32, 51, 4, 76, 24, and 75, and those can be interpreted as the prioritized therapeutic interventions. The importance rating results on those 8 clusters confirmed by the Ward's method are presented in Table 2. The importance rating by cluster showed that Cluster 8 (Counselor Self-Awareness and Regulation) had the highest mean *M* = 5.77, followed by Cluster 4 (Conceptualization of Suicide Crisis) with *M* = 5.63 and Cluster 1 (Securing Safety) with *M* = 5.59.

**Table 2:** Importance rating mean for each cluster on therapeutic interventions.

**Cluster**	**Average rating of importance**	***SD***
Cluster 1: Securing Safety	5.59	0.70
Cluster 2: Active Advocacy for Client	5.27	0.85
Cluster 3: Coping Skills Training	5.27	0.91
Cluster 4: Conceptualization of Suicide Crisis	5.63	0.73
Cluster 5: Emotional Identification and Validation	5.54	0.58
Cluster 6: Empowerment	5.15	0.92
Cluster 7: Counselor Self-disclosure	4.91	1.08
Cluster 8: Counselor Self-awareness and Regulation	5.77	0.86

## Discussion

The present study used concept mapping to analyze the experienced counselors' perception of effective therapeutic interventions. The advantages of this methodology include that it fits well with an explorative stage of research questions yet to own confirmed theoretical background while minimizing the researcher's subjective viewpoint (64). Concept mapping may appear identical to qualitative methodology as it explores the participants' potential awareness; however, it is based on a statistical method for objective results (65, 66, 69, 70) as it allows quantitatively substantiated participants' conceptualizations.

The present study explored and examined 77 therapeutic interventions of suicide crisis counseling through experienced counselors with a minimum of 5 years of counseling experience and 10 counseling sessions with the clients of suicide attempts in South Korea. Through the analysis, the study discovered eight clusters on the concept map and demonstrated three important themes regarding the therapeutic interventions of suicide crisis counseling. Experienced Korean counselors practiced active and instructional intervention (Cluster 1: Securing Safety, Cluster 2: Active Advocacy for Client, Cluster 3: Coping Skills Training), understood the clients' difficulties in emotional expression and had patience in waiting, and involved with the clients' painful feelings (Cluster 5: Emotional Identification and Validation). Additionally, they had high involvement therapeutic relationships with their clients through self-disclosure and expressions for self-worthiness in their clients (Cluster 6: Empowerment, Cluster 7: Counselor Self-Disclosure, Cluster 8: Counselor Self-Awareness and Regulation).

### Active and Instructional Intervention

Cluster 1 (Securing Safety), Cluster 2 (Active Advocacy for Client), and Cluster 3 (Coping Skills Training) are situated on the right side of the concept map. This indicates that they all show common characteristics of a counselor's active and instructional intervention. The counselors in Cluster 1 reported receiving promises not to commit suicide from their clients and showed firm expression against suicide as an effective therapeutic intervention. This may appear authoritative and instructional, but it aligns with successful factors in previous research that effective counseling techniques in Korea include providing advice and instructional intervention (26, 28, 29, 32).

Cluster 3 (Coping Skills Training) indicated that the counselors tended to actively educate alternative skills such as finding alternative behaviors to suicide, effective interpersonal competency skills, and reduction of self-destructive behaviors as the therapeutic intervention. The perception of considering active education as a therapeutic intervention is also seen in Korean suicide risk factors. In the studies on suicide group with the comparison group, self-injuries, previous suicide attempts, alcohol problems, financial hardship, failure of a business, interpersonal issues with domestic partners, and other factors related to a heightened stress level were related to suicide (23). The interventions to help gain some of these proficiencies as interpersonal skills, to reduce self-destructive behaviors (e.g., alcohol abuse, dangerous sexual acts) and self-injury, appear to be effective therapeutic interventions.

Cluster 2 (Active Advocacy for Client) is located, overlapping with Cluster 1 (Securing Safety) and Cluster 3 (Coping Skills Training). This portrays the higher chances of simultaneous occurrence or more relatedness among those three clusters. When the counselors continue with the interventions of Cluster 1 (Securing Safety) and Cluster 3 (Coping Skills Training), they actively take action to advocate for their clients. Such interventions may have been influenced by the Korean counseling environment. The intervention of counselors seeking assistance from the institutions and intervention of discussing the appropriate time and method of counseling termination with the client are not reported by the suicide crisis intervention list (36–38) in western culture. The experienced Korean counselors' additional effort in these areas can be viewed as providing additional services to the clients in the absence of sufficient insurance services related to suicide crisis cases in Korea.

The most important intervention was rated for Cluster 1 (Securing Safety); providing the emergency contact number and checking for the client's safety in an emergency and helping to calm down belonged to this cluster. In particular, most participants in the study reported that they provide their personal phone numbers, demonstrating active involvement, yet it also may provide an insight into the underdeveloped counseling system in Korea. As mentioned earlier, Korea still has a short history of counseling, and the effective connections between hospital/police/emergency medical personnel are still weak. Hence, counselors often offer themselves as the emergency contact persons and perceive the need to actively handle emergencies themselves as a therapeutic intervention. The development of efficient connections in those three organization types should continue. Finding the means to check for the clients' safety and proper education on safety measures to handle dangerous clients is necessary for the interim.

### Patience in Waiting for Emotional Expression Based on Empathy

According to Cluster 5 (Emotional Identification and Validation), the experienced counselors in South Korea perceived patience waiting for their clients to be able to express emotions and validating such emotions to be a highly therapeutic intervention. For instance, the participants reported understanding and sensitively responding to the clients' major emotions (e.g., extreme frustration for feeling alone) as a therapeutic intervention and had a tendency to look into co-events occurring in repeated suicidal impulses and the suicide attempt processes. They also assisted their clients by encouraging them to specifically express negative feelings, help to utilize pictures, mental images, and other various tools to express, all of which were perceived as therapeutic interventions. They reported understanding and enduring even uncooperative clients and validating their feelings and empathizing with the clients' continuous thoughts on death as an effective therapeutic intervention.

Those interventions perceived by the counselors are very unique interventions rarely listed as interventions by diagnosis and intervention capacities for suicide crisis (38, 71) recommended and developed by the Assessing and Managing Suicide Risk (AMSR), American Association of Suicidology (AAS). The results portray that many Korean clients have shown difficulties in expressing their emotions, very likely rooted in Confucian-based philosophy and communitarianism; yet assisting with their expression of emotions was considered effective counseling technique. This supports the results of a previous study that found higher effectiveness with emotional expressions as the focal point in counseling (27). Other research findings also supported that effective Korean counselors tended to understand the clients' verbal awkwardness in expressing their own problems and feelings and therefore patiently waiting for a longer time (26, 32). A study on suicide crisis of adolescent group similarly revealed that the counselors with more verbal responses acknowledging the clients' feelings and reflecting their emotions had a higher success rate in agreed termination of counseling sessions (72). Future Korean suicide crisis counseling education warrants exploring verbal inexpression nature in clients along with finding how counselors can assist with language, mental images, for instance, to encourage more verbal expressions. Also, education on staying with the devastation and holding the client's negative emotions is necessary. It is noteworthy to focus on this aspect of counseling intervention factor especially when emotional involvement is often discouraged in practice in a crisis counseling setting (73, 74).

### High Involvement

Situated on the lower left side of the two-dimensional concept map, Cluster 6 (Empowerment), Cluster 7 (Counselor Self-Disclosure), and Cluster 8 (Counselor Self-Awareness and Regulation) indicate that experienced Korean counselors are engaged in deep, therapeutic relationships with their clients with high involvement. Their tendency to believe that expressing to the client as a meaningful and valuable being is an important intervention that is aligned with the Interpersonal Theory of Suicide (IPTS) described earlier. As seen in the empirical studies in previous research (12–16), abrupt socioeconomic changes in Korea are believed to have resulted in weakened social integration, a sense of isolation and burdensomeness to others in individuals, and such interpersonal factors contribute to the nation's suicide rate (7, 10, 11). Within Cluster 6 (Empowerment), counselors treat their clients with values and support with a positive attitude along with leading to cooperation after a steady rapport is established; it is identical to the findings of the western therapeutic intervention (36, 37). However, when the professional boundaries are violated, the counselors further take actions such as first accepting and supporting by being supportive partners and re-nurture upon developmental phases of the clients. Focusing on Cluster 7 (Counselor Self-Disclosure) provides another meaningful insight; Korean counselors' disclosure of their human feelings by the client's suicide impulse/attempts and expressing the client's unique values are not witnessed as effective interventions in western societies. This is to say that Korean counselors readily provide themselves as a “shelter” to their clients while enabling them to believe their value to others, perceived as an effective therapeutic intervention.

The highest score in the importance rating was for Cluster 8 (Counselor Self-Awareness and Regulation), which displays another interesting intuition. Experienced Korean counselors understood that owning a high level of involvement with the client's emotional needs inevitably was linked to countertransference and exhaustion in them. Nonetheless, they were very well aware of the importance of managing and controlling their feelings well. To avoid negative intervention caused by their own emotions, recognizing them, enduring negative and suppressing feelings, and being self-aware and regulating were considered important therapeutic interventions. Cluster 7 (Counselor Self-Disclosure) and Cluster 8 (Counselor Self-Awareness and Regulation) located near Cluster 6 (Empowerment) further denote that applying a therapeutic relationship means high involvement with disclosing self-emotions for creating a true relationship. A study of the grounded theory method revealed that inexperienced counselors initially were fearful of the client's suicidal behaviors; however, after becoming more competent at recognizing their own feelings and controlling them, their coping skills significantly increased (75). Such a high important rating score result for Cluster 8 (Counselor Self-Awareness and Regulation) signifies that self-awareness in negative, overwhelming feelings from their clients must well be controlled. This, in turn, can help reduce the clients' sense of burdensomeness and recover the sense of belongingness through a healthy therapeutic relationship with their counselor.

### Implications

First, a final set of 77 statements of therapeutic intervention drawn from the present study presents a useful list that future suicide crisis counseling education may utilize in Korea. In sum, experienced Korean counselors overall perceived patiently aiding with emotional expressions, offering self as a nurturer, and self-disclosing for creating a true, therapeutic relationship as effective therapeutic interventions. Such interventions were often qualitatively different from the ones found in the western studies in suicide crisis counseling; this study realistically offers Korea-specific practical counseling components to its clients in the related area. In addition, interventions that had six or higher scores in importance rating (Statement 28, 59, 38, 31, 32, 51, 4, 76, 24, 75) may be considered as more urgently needed interventions that should be implemented quickly.

Second, the present study uniquely studied how experienced Korean counselors perceived therapeutic interventions in groups. Through the study, they perceived therapeutic interventions with eight clusters. Speculating the locations of each cluster on the concept map, more closely related clusters should be taught for educational purposes in the suicide crisis counseling curriculum in the future. For instance, Cluster 1 (Securing Safety) and Cluster 3 (Coping Skills Training) near Cluster 2 (Active Advocacy for Client) together indicate that teaching advocacy concerning clients' rights can be implemented along with educating for clients' safety and coping skills training. Likewise, when the counselors empower (Cluster 6: Empowerment) their clients through a therapeutic relationship, making sensible use of self-disclosure (Cluster 7: Counselor Self-Disclosure) and how to effectively manage counselors' own negative feelings (Cluster 8: Counselor Self-Awareness and Regulation) can be co-trained for more in-depth quality in suicide crisis counseling.

### Limitations and Future Directions

The study limitations and suggestions for future studies are as follows. First, the present study relied on the experienced counselors' self-reports only on the clients with “suicide attempts with suicide intention” as any clinical scale was lacking in the Korean suicide crisis counseling setting. Yet, an objective clinical scale for the measurement of suicide crisis in at-risk clients will be beneficial for future studies. Second, the present study examined the perception of the experienced counselors on effective therapeutic intervention in suicide crisis counseling, which may be different from the perceptions of the clients. Therefore, future studies may investigate effective therapeutic interventions perceived by the clients and follow up with any observable episodes, such as a reduced number of suicide ideations or attempts to measure the results on the clients' side. Third, this study considered Korea's short counseling history and defined experienced counselors as “with a minimum of 5 years of counseling in the field and 10 completed sessions of counseling in a suicide crisis.” It must be noted that experienced counselors do not always reflect that they are “experts” in the field (76). Future studies can adjust and meet more fine definitions for suicide crisis counseling experts as their study participants in Korea and abroad. Fourth, this study had enough participants as concept mapping methodology requires; however, it is still recommended that the results be interpreted with caution and be generalized from the selected participants in this study.

## Conclusion

The present study examined experienced South Korean counselors' perceptions on therapeutic interventions in preventing or delaying completed suicide using concept mapping methodology and provided a visual representation of its results. A total of 77 final statements from 15 study participants were extracted and their perceptions were grouped into 8 major clusters on a two-dimensional model. The interpretations of cluster locations and importance rating results of this study are expected to contribute as an educational foundation for suicide crisis intervention programs for counselors in the field. A broader understanding of the necessities related to the clients' needs, beyond the Korean client population, in suicide crisis counseling and strengthening suicide crisis intervention competence in counselors for ultimate suicide prevention are further encouraged.

## Data Availability Statement

The original contributions presented in the study are included in the article/supplementary material, further inquiries can be directed to the corresponding author.

## Ethics Statement

The studies involving human participants were reviewed and approved by Institutional Review Board of Seoul National University (IRB No. 1906/003-015). The patients/participants provided their written informed consent to participate in this study.

## Author Contributions

AP and DK: conceptualization and methodology. AP, DK, and HS: validation and writing—review and editing. AP: formal analysis, investigation, visualization, data curation, and project administration. AP and HS: resources and writing—original draft preparation. DK: supervision. All authors have read and agreed to the published version of the manuscript.

## Funding

This work was supported by the Ministry of Education of the Republic of Korea and the National Research Foundation of Korea (NRF-2020S1A3A2A02103411).

## Conflict of Interest

The authors declare that the research was conducted in the absence of any commercial or financial relationships that could be construed as a potential conflict of interest.

## Publisher's Note

All claims expressed in this article are solely those of the authors and do not necessarily represent those of their affiliated organizations, or those of the publisher, the editors and the reviewers. Any product that may be evaluated in this article, or claim that may be made by its manufacturer, is not guaranteed or endorsed by the publisher.
